# Clinicopathological Diversity of Canine Mammary Gland Tumors in Sri Lanka: A One-Year Survey on Cases Presented to Two Veterinary Practices

**DOI:** 10.3390/vetsci5020046

**Published:** 2018-04-27

**Authors:** Harsha Ariyarathna, Niranjala de Silva, Danielle Aberdein, Dayananda Kodikara, Manjula Jayasinghe, Ranjith Adikari, John S. Munday

**Affiliations:** 1 School of Veterinary Science, Massey University, Palmerston North 4442, New Zealand; h.ariyarathna@massey.ac.nz (H.A.); d.aberdein@massey.ac.nz (D.A.); 2 Faculty of Veterinary Medicine and Animal Science, University of Peradeniya, Peradeniya 20400, Sri Lanka; niranjalad@yahoo.com (N.d.S.); chamivet@gmail.com (M.J.); adikari04@yahoo.com (R.A.); 3 New Animal Clinic, 132/B, S. De. S. Jayasinghe Mawatha, Kohuwala, Nugegoda 10250, Sri Lanka; dskodikara132@gmail.com

**Keywords:** canine, mammary gland tumors, malignant tumors, Sri Lanka

## Abstract

Mammary gland tumors (MGTs) are one of the most common neoplasms among dogs in Sri Lanka. However, the clinicopathological diversity of MGTs in Sri Lanka is largely unknown, impeding accurate diagnosis and effective treatment of the disease. The present study investigated the clinicopathological features of MGTs in 74 dogs presented to two veterinary practices in Sri Lanka treated surgically, over a one-year period. Information regarding the patient signalment, clinical presentation, and reproductive history were collected, and each neoplasm was examined histologically. Forty-one (54.4%) dogs were primarily presented for mammary neoplasia, while a MGT was an incidental finding in 33 (44.6%) dogs. The majority of tumors were histologically malignant (n = 65, 87.8%), and 18 malignant tumor sub-types were identified. A significantly higher proportion of malignant tumors were large (>3 cm diameter) and observed in inguinal mammary glands. Nulliparous (n = 42, 55.3%) dogs predominated in the group, and the mean age of MGT diagnosis was 8.0 ± 2.41 years. The present study identified tumor location and size to be predictive of malignancy. A high histological diversity of MGTs was observed. Overall, the present findings emphasize the necessity of improving awareness of MGTs among Sri Lankan clinicians as well as dog owners.

## 1. Introduction

As female reproductive hormones promote mammary carcinogenesis, MGTs are the most common neoplasm among intact dogs [1,2]. Ovariohysterectomy (OHE) performed at an early age minimizes the prolonged exposure of mammary tissues to reproductive hormones, and thereby reduces the risk of mammary neoplasia [3]. Specifically, rates of MGTs in dogs that undergo OHE prior to first estrus are around 8%, but this rate increases to 26% in dogs that undergo OHE between the first and second estrus [4]. Consequently, the incidence of canine MGTs is decreasing in the regions of the world where OHE is routinely performed at an early age [5]. Sri Lanka is a south Asian country where spaying of dogs at an early age is not a common practice [6]. Dog spaying in Sri Lanka is mostly conducted during mass de-sexing programs that are generally done on an opportunistic basis [6,7]. Therefore, most of the dogs in Sri Lanka are either intact or have been spayed at an older age, which predisposes them to mammary neoplasia. In fact, MGTs are one of the most common neoplasms of dogs in Sri Lanka, and are an important cause of mortality in this population [8].

Given the importance of this disease in Sri Lanka, it is desirable to improve the diagnostic, prognostic, and therapeutic aspects of canine MGTs. To achieve this, a basic understanding of the clinicopathological diversity of MGTs in the Sri Lankan dogs is essential. Therefore, the primary aim of the present study was to determine the different clinicopathological aspects of MGTs presented to two veterinary practices in Sri Lanka during a one-year period. It was considered possible that MGTs in Sri Lankan dogs would show some features that are different to MGTs in dogs elsewhere in the world. These features could be a useful guide for Sri Lankan veterinary pathologists and clinicians to inform—and possibly modify—their current diagnostic and therapeutic approaches, allowing a more accurate diagnosis and more effective treatment of canine MGTs in Sri Lanka dog.

A knowledge of possible risk factors for canine MGTs is important to help developing effective strategies to minimize the incidence of these neoplasms. Previous studies have suggested that dogs older than 7 years, small-sized dogs, obese dogs, and dogs that were spayed later in life but still nulliparous, are at increased risk for MGTs [2,9]. However, the relative impact of these risk factors appears to be variable within different regions of the world. Therefore, as a secondary objective, we determined the age, breed, body condition score, reproductive status, and parity of the dogs with MGTs to identify the common profile of affected dogs in Sri Lanka.

The findings of the current study will provide a basic understanding about the clinicopathological diversity of MGTs in Sri Lanka. By determining the common characteristics of dogs with MGTs, it may be able to identify some of the likely risk factors of canine MGTs in Sri Lanka. To the authors’ knowledge, there are no published studies describing MGTs in dogs in Sri Lanka.

## 2. Materials and Methods

Sample collection: Samples for the present study were obtained from the Veterinary Teaching Hospital (VTH), Faculty of Veterinary Medicine and Animal Science, University of Peradeniya, and a private veterinary practice in Colombo (VPC), Sri Lanka. All dogs that presented to these clinics between June 2016 and June 2017, with one or more spontaneous MGTs that were treated by surgical excision, were included in the study. The primary clinicians that attended to the MGT cases kindly provided us with the patient records and surgically excised mammary tumors collected in 10% neutral buffered formalin. Full owner consent was obtained before the sample collection.

The following information was determined from the provided patient records: primary complaint, breed, age, body condition score, age at neutering, parity, general clinical exam findings, and information regarding any prior investigations related to the presenting MGTs. According to the tumor diameter specified in the patient records, tumors were classified as T1 (≤3 cm in greatest dimension), T2 (tumor >3 cm but <5 cm in greatest diameter), or T3 (tumor >5 cm in greatest diameter), following the World Health Organization guidelines as applied by Sorenmo et al. (2007) [5]. Other gross pathological features of the tumors: adherence of tumor mass to the underlying tissues and ulcerations on the skin overlying the tumor, were also extracted from the patient records.

Histopathology and tumor classification: The formalin-fixed MGTs received from the primary clinicians were further processed in the histopathology laboratory of the VTH. Briefly, tumors were dehydrated in a gradient of alcohol, embedded in wax, processed in to thin sections (3-µm), stained with hematoxylin and eosin, and examined microscopically. When multiple tumors were present in a single dog, only the tumor with the greatest diameter was considered for histological examination. Immunohistochemistry for smooth muscle actin (SMA) was performed to evaluate a myoepithelial origin of the neoplastic cells using an anti—α SMA antibody (Sigma-Aldrich, St. Louis, MO, USA), following the standard protocols using vascular smooth muscles as the positive control and cardiac muscle as the negative control. Mammary tumor classification was performed according to the definitions in the 2011 classification proposed by Goldschmidt and colleagues [10]. Histological malignancy of the tumors was determined following the criteria described in the 2011 Goldschmidt classification: tumor type, nuclear and cellular pleomorphism, mitotic index, presence of randomly distributed areas of necrosis within the neoplasm, peri-tumoral and lymphatic invasion, and presence of intra-tumoral inflammatory cell infiltration [10]. All carcinomas except inflammatory carcinomas were graded according to the guidelines provided by Pena et al. (2012) [11]. Briefly, tubule formation, nuclear pleomorphism, and mitotic counts were considered and rated on a scale from 1 to 3. The scores for each category were added together and the total scores were used to determine the histological grades for each tumor. In heterogeneous carcinomas, tubular scoring was assessed in the most representative malignant area. In complex and mixed tumors, the percentage of tubular formation was scored considering only epithelial areas, and nuclear pleomorphism was evaluated in all the malignant components.

Statistical analysis: All statistical tests were performed using add-on for Excel/XLSTAT software (Version 2017.4) (Addinsoft, New York, NY, USA). The single proportion test was used to compare the malignant tumor proportions in the right and left inguinal mammary glands respectively. Differences were considered significant if the calculated *p* values were <0.05.

A chi-square test was performed to determine whether the malignant mammary tumors were equally distributed among the thoracic, abdominal and inguinal mammary glands. The chi-square test was followed by the Marascuillo process, to identify the glands which had a significantly different proportion of malignant mammary tumors compared to the other glands. The number of malignant MGTs in each anatomical location was compared with the MGTs in other locations in a pair-wise manner using a calculated absolute and critical value for each pair. The difference was considered significant if the calculated absolute value was greater than the critical value. The same statistical methods were used to investigate differences in the distribution of malignant mammary neoplasms among T1, T2 and T3 tumor size categories. 

## 3. Results

### 3.1. Clinical Characterization of Dogs with Mammary Gland Tumors

Seventy-four dogs with MGTs were included in the study. Thirty-six (48.6%) of them were from Veterinary Teaching Hospital (VTH), Peradeniya, and 38 (51.4%) were from the veterinary practice in Colombo (VPC).

Out of the 74 dogs included in the study, 41 (55.4%) dogs were presented primarily seeking veterinary care for mammary neoplasia. Among these, 11 dogs had tumors that had been previously diagnosed as benign MGTs by cytology. In addition to a MGT, reduced appetite and lethargy were secondary complaints in 10 (13.5%) and 17 (23.0%) dogs respectively. In 33 (44.6%) dogs, mammary neoplasia was detected during a clinical examination when the dog was presented for an unrelated complaint. These dogs were presented for veterinary care primarily due to reduced appetite (n = 19, 25.7%) or lethargy (n = 14, 18.9%). Interestingly, the owners of 9 (12.1%) of the dogs admitted that even though they had noticed the mammary masses in their dogs prior to the clinical exams, they did not seek veterinary care specifically regarding them, assuming that the masses were harmless. 

During clinical examination of the 74 dogs, lymphadenopathy was identified in 40 (54.1%) dogs. According to the clinical records, lymphadenopathy was detected in inguinal lymph nodes alone in 10 (13.5%) dogs, while both inguinal and popliteal lymph nodes were concurrently enlarged in 15 (20.3%) dogs. The affected lymph nodes were not specified in 15 (20.3%) dogs. Cytological examination of fine needle aspirates from the enlarged lymph nodes was performed in only 4 dogs and none of the aspirates were reported to contain neoplastic cells. Further, concurrent with lymphadenopathy, dyspnea or pyrexia was detected during clinical examination in 16 (21.6%) and 14 (18.9%) dogs respectively. Lateral thoracic radiographs had been taken in six dogs and evidence suggestive of pulmonary tumor metastasis was observed in 3 dogs. Weight loss was recorded in 7 out of the 30 dogs for which previous weight records were available. Two dogs were diagnosed with pyometra in addition to mammary neoplasia. Overall, 54 dogs, which accounted for approximately 75% of the group, were systemically ill at the time of presentation.

Thirty nine of the 74 dogs presented were mixed breed dogs which represented approximately half of the group (50%). German shepherd was the most common pure dog breed (n = 21, 28.4%), followed by dachshund (n = 2, 2.7%). Single cases of following breeds were also observed: Boxer, Fox Terrier, Japanese Spitz, Pomeranian, Tibetan Terrier, Cocker Spaniel, English Springer Spaniel, Pekingese, Doberman, Dalmatian, Great Dane, Labrador Retriever, Rottweiler and Rhodesian Ridgeback.

The overall mean and median ages of dogs with MGTs were 8.0 ± 2.41 years and 8.0 years respectively. The age of the dogs with MGTs was further analyzed separately in 4 categories, namely: 0–4 years, 5–8 years, 9–12 years and ≥13 years. The most frequently represented age category was 5–8 years, which included 41 (55.4%) dogs. There were 26 (35.1%) dogs in the 9–12 years category. The categories 0–4 years and ≥13 years, which included the youngest and oldest dogs, were less frequently represented as there were only 4 (5.4%) and 3 (4.0%) dogs in those categories respectively. The mean age of the dogs with benign and malignant MGTs were 6.7 ± 1.66 years and 7.9 ± 2.15 years respectively. There was no significant difference between the age of the dogs with benign tumors and malignant tumors.

Primary clinicians had used the 1–5 body condition scoring system described by Eastland-Jones et al. (2014) [12]. Accordingly, the body condition of the majority of dogs was BCS 3 with 42 (56.8%) dogs in this category. BCS 2 and BCS 4 categories included 15 (20.6%) and 13 (17.6%) dogs respectively, while the BCS 5 group was the least represented group of all (n = 4, 5.4%).

Reproductive status of the dogs with MGTs is summarized in Table 1. The majority of dogs were intact, while the remainder had been spayed at varying ages.

Forty-two dogs (55.3%) included in the study were nulliparous. Others had reportedly whelped once (n = 12, 21.1%), twice (n = 18, 23.7%) or three times (n = 4, 5.3%). Contraceptives had not been used to prevent pregnancy in any of the dogs included in the study.

### 3.2. Gross Pathological Characterization of the Mammary Gland Tumors

Tumors were detected in a single mammary gland in 53 (71.6%) dogs, while 21 (28.3%) dogs had tumors in multiple mammary glands. The distribution of the mammary tumors among the mammary glands is summarized in Table 2. Inguinal mammary glands were most often affected: 46 (62.1%) dogs had tumors in these glands. Surprisingly, a significantly higher (*p* < 0.001) number of dogs had tumors in the left inguinal gland (n = 42, 56.8%) than the right (n = 4, 5.4%). No such significance was detected in MGTs in the thoracic or abdominal mammary glands. Nine inguinal MGTs were associated with another mammary tumor in a different location, while 4 inguinal MGTs were associated with multiple tumors in other glands.

The size of the MGTs is summarized in Table 3. Overall, 31 dogs had T2 tumors, 25 had T3 tumors, and 18 had T1 tumors. Dogs which were primarily presented for mammary neoplasia had either T2 (n = 18) or T3 (n = 25) tumors, but none had T1 tumors. The MGTs which were incidentally detected in a clinical exam of a dog that was presented for unrelated complaints were either T1 (n = 18) or T2 (n = 15). 

Ulceration of the skin overlying the tumor was observed in 20 (27.0%) dogs, while 15 (20.3%) tumors were fixed to the underlying tissues.

### 3.3. Histological Characterization of the Mammary Gland Tumors

**Figure 1:**
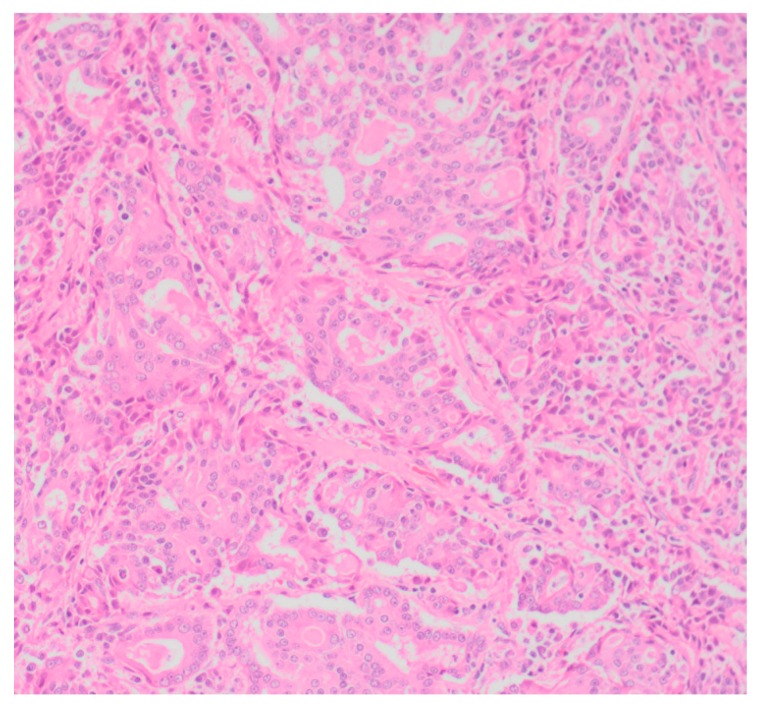
Tubular carcinoma (Grade III), mammary gland, canine (low power).

**Figure 2:**
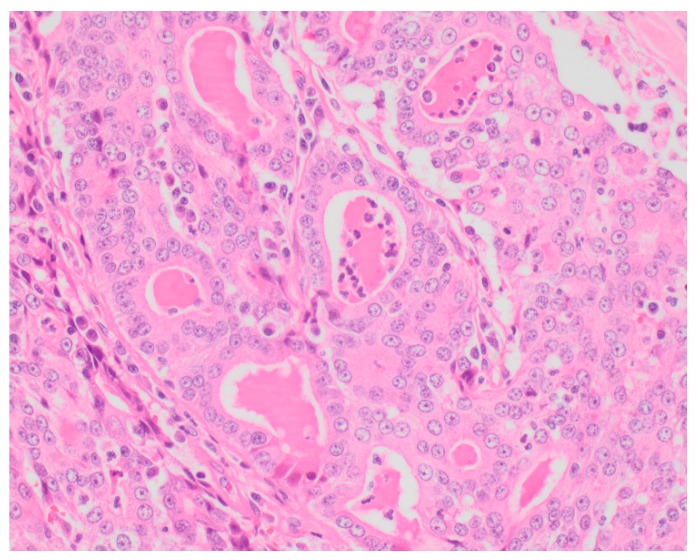
Tubular carcinoma (Grade III), mammary gland, canine (high power). Highly pleomorphic neoplastic cells exhibiting multiple cellular and nuclear criteria of malignancy.

Sixty-five (87.8%) of the tumors examined histologically were classified as malignant, while 9 (12.2%) were classified as benign. All MGTs histologically identified as malignant included at least three cellular or nuclear criteria of malignancy (Figure 1 and Figure 2). Histological evidence of peri-tumoral (n = 6) and lymphatic (n = 4) invasion was identified in 10 malignant tumors, while randomly distributed areas of necrosis within the neoplasm were observed in 16 malignant tumors.

Using the histologic classification of malignant MGTs, tumors were identified in three categories: carcinomas, carcinomas-special types, and sarcomas (Table 4). There were 9 sub-types of carcinomas, 6 sub-types of special carcinomas and 3 sub-types of sarcomas. The carcinoma sub-types, simple carcinoma (n = 13, 17.6%) and mixed-type carcinoma (n = 10, 10.8%), were the most frequent sub-types in the carcinoma group. Thirteen simple carcinomas included 5 tubular carcinomas, 7 tubulo-papillary carcinomas, and 1 cribriform carcinoma. In addition, single cases of ductal carcinoma, anaplastic carcinoma, and carcinoma-spindle cell variant were identified in the carcinoma group. Adenosquamous carcinoma (n = 8, 10.5%) was the most frequent special type carcinoma, while hemangiosarcoma (n = 2, 2.7%) was the most frequent type of sarcoma. Simple adenoma (n = 3, 4.0%) was the most frequent benign MGT sub-type, while single cases of fibroadenoma and mixed benign tumor sub-types were observed. Immunostaining using antibodies against smooth muscle actin (α-SMA) was present in 3 MGTs interpreted to be complex carcinomas and in 1 neoplasm that was classified as a malignant myoepithelioma. The absence of immunostaining was used to support a classification of mammary gland fibrosarcoma in one case.

Intra-tumoral inflammatory cell infiltrates were observed in 21 malignant tumors; moderate intra-tumoral cell infiltration was identified in 11 tumors, and it was low and marked in 6 and 4 tumors respectively. Sixty carcinomas were graded. Of the sixty, 24 were classified as grade I, 19 were grade II, and 17 were grade III carcinomas.

All the dogs that were presented primarily due to MGTs had neoplasms that were classified as malignant, and all the benign tumors included in this study had been detected incidentally during clinical examination. No clinical features allowed definitive determination between malignant and benign neoplasms. However ability to associate malignancy with a clinical feature in this study was impaired by the small number of dogs with dog benign MGTs. In all the dogs with multiple MGTs, the tumors with the greatest diameters were malignant. In addition, all the tumors with surface skin ulcerations, and those which were fixed to the underlying tissues, were malignant.

The tumor location was predictive of malignancy with a significantly higher proportion of malignant MGTs developing in the inguinal mammary glands than in the thoracic and abdominal mammary glands (Table 2). Regarding the tumor size, the proportions of malignant T2 or T3 tumors were significantly higher compared to the proportion of malignant T1 tumors (Table 3). Thus, T2 or T3 tumors are more likely to be malignant than T1 tumors. However, there was no significant difference between the proportions of malignant T2 and T3 tumors. This indicates that the further differentiation of large MGTs in to T2 or T3 categories does not provide any additional advantage when predicting malignancy. 

## 4. Discussion

The present study reports a systematic evaluation of the clinicopathological features of MGTs in 74 Sri Lankan dogs. Within these dogs, 88% of the MGTs were histologically classified as malignant. This proportion of malignant MGTs is higher than the 40–50% of MGTs reported to be malignant in studies conducted in United States of America (USA) [13], Canada [14], Japan [15], and Mexico [16]. However, the proportion of malignant tumors observed in Sri Lankan dogs was similar to the rates reported from India (83%) and Brazil (86%) [17,18]. The reasons for a higher proportion of malignant MGTs in dogs in Sri Lanka, Brazil and India are unknown. It is possible that the malignant MGTs were over-represented in these countries as a consequence of frequent exposure of the dogs to carcinogens which may not be present in countries such as the USA, Canada, Japan and Mexico. However, it is also possible that malignant MGTs were over-represented in the developing countries as a consequence of under-detection of benign MGTs. Benign MGTs are mostly incidental clinical exam findings, and not the primary concerns of the dog owners [19]. The incidental detection of benign MGTs during a clinical examination may be less likely to occur in developing countries because dog owners may seek veterinary care less frequently compared to owners in more developed countries. It appears likely that owners may not seek veterinary advice unless they observe a rapidly growing ulcerated mammary gland mass. Such masses are much more likely to be malignant tumors [20]. This is supported by the observation in the present study that all dogs that were presented for a mammary gland mass had malignant tumors, while all the benign tumors observed in this study were from dogs that had presented to the veterinarian for a reason not related to the MGT. The high percentage of MGTs that were malignant at the time of presentation in Sri Lanka suggest that veterinarians should be aware of this disease, and consider mammary neoplasia as a serious health problem among Sri Lankan dogs. In this study, when there were multiple mammary tumors in a single dog, only the tumor with the greatest diameter was considered for histological analysis due to the financial limitations. The preferential examination of the largest neoplasm could have contributed to the higher proportion of MGTs being malignant in this study. 

Nine malignant MGTs included in the present study had been previously diagnosed as benign, using cytology. The discrepancy could be either due to the limited capability of cytology to differentiate benign from malignant tumors [13] or possible benign to malignant transformation which had occurred during the lapse of time between the initial diagnosis and the second examination [20,21]. This observation suggests that pathologists should be cautious when classifying a MGT as benign solely on cytology. Additionally, it suggests that benign tumors should be carefully monitored for evidence of progression to a malignant neoplasm. It is noteworthy that nine dog owners who had observed the MGTs in their dogs had not considered them as conditions requiring veterinary care. This indicates that some Sri Lankan dog owners may not to be sufficiently aware of the adverse consequences of MGTs, and emphasizes the necessity of improving awareness on MGTs among the Sri Lankan dog owners. 

In the present study, the majority of MGTs were detected in the inguinal mammary glands. This distribution is consistent with the findings of many previous studies [22,23,24]. The frequent involvement of the inguinal glands is attributed to their abundant tissue mass and prolonged secretory activity, compared to other glands [20]. However, unlike in previous studies, our results show significantly higher involvement of the left inguinal gland compared to the right. As both left and right inguinal glands have been previously reported to be affected at equal rates [16,20], the marked left gland involvement observed in the present study is difficult to explain. 

In this study, the proportion of malignant MGTs in the inguinal glands was significantly higher than the proportions of MGTs in the thoracic or abdominal MGTs. This has not been described in previous studies, and the reason for a higher proportion of malignant inguinal MGTs in the present study is unknown.

The results of the present study suggest that tumors which have a diameter >3 cm are more likely to be malignant. This is consistent with the findings of previous studies. A retrospective study conducted by Philibert and colleagues confirmed that dogs with tumors >3 cm in diameter to have decreased overall survival compared to the dogs with tumors <3 cm in diameter [25]. In another study, tumor size of >3 cm diameter was correlated with the factors indicating poor prognosis, such as loss of hormone receptors or higher proliferation index [5]. However, tumor size alone does not confirm the malignancy of a mammary tumor, and histological examination is essential for confirmation. In the present study, all the tumors with surface skin ulcerations, and tumors which were fixed to the underlying tissues, were malignant, indicating that these features could also be predictive of malignancy. Previous studies also indicate that malignant MGTs which are usually large in size are more likely to develop ulceration, due to more frequent contact with the rough surfaces compared to small size benign tumors [20]. Since malignant neoplasms invade or infiltrate surrounding muscle, nerve, blood vessels, and connective tissues, they are also more likely to become fixed to the underlying tissues [20]. 

An interesting feature regarding the reproductive histories of the dogs in the present study was that over half of the dogs were nulliparous. A recent study conducted in Switzerland in 2018 confirmed that nulliparous dogs are at a significantly higher risk for developing mammary tumors compared to multiparous dogs [26]. The elevated risk was attributed to the higher frequency of pseudopregnancy and estrus in nulliparous dogs than multiparous dogs. As both pseudopregnancy and estrus increase the production of female reproductive hormones, the mammary gland tissues of nulliparous dogs may be exposed to greater amounts of female hormones than the mammary gland tissues of multiparous dogs [26]. While the results of the present study suggest that nulliparous dogs in Sri Lanka may be at similar higher risk for MGTs, it has to be noted that the proportion of dogs in the studied populations that are nulliparous is unknown. Therefore, it is possible that the high proportion of dogs with MGTs that were nulliparous in the study was simply due to the high proportion of dogs in Sri Lanka that are nulliparous. 

The histological diversity of the malignant MGTs included in the present study was high. In fact, out of the 23 malignant MGT sub-types listed in the Goldschmidt classification, 18 were reported in the present study. A recent prospective study conducted in Italy confirmed the prognostic significance of the Goldschmidt classification [27]. Given the high histological diversity revealed by the present results, Sri Lankan pathologists should be able to accurately differentiate tumor sub-types in order to provide reliable prognostic information. In this paper we used α-SMA to help differentiate between complex and simple carcinomas. In addition, the same antibody was used to help differentiate the myoepithelial origin of a malignant myoepithelioma, and to exclude myoepithelial origin within a mammary gland fibrosarcoma. However, definitive differentiation of myoepithelial cells was not possible using α-SMA alone. Instead, it is currently recommended that p63 or a panel of antibodies be used to differentiate between these tumor types. In the present study, only α-SMA was used to reproduce the likely situation in developing countries in which p63 is not often available, and clients are unlikely to be able to afford a panel of immunostains. Interestingly, one of the simple mammary carcinomas reported in the present study co-existed with a cutaneous mast cell tumor. Therefore, apart from being classified as a mammary carcinoma, it was identified as a collision tumor referring to the mixed presentation. Collision tumors are a type of a mixed tumor with 2 foci of neoplasia which develop adjacent to one another, yet remain separate [28]. These tumors are rare, and there is minimal information regarding treatment recommendations and outcome for animals [28].

A majority of affected dogs in the present study were 5–8 years old, and the mean age of the diagnosis of mammary neoplasia was 8.0 ± 2.47 years. The mean age reported from Sri Lanka is comparatively lower to the age of diagnosing mammary neoplasia in dogs reported from Sweden (9.33 years) [29], Slovenia (10 years), Turkey (10.3 years) [23], Canada (11 years) [14], Brazil (11.6 years and 12 years) [30,31], Mexico (9–12 years) [16] and Czech Republic (13 years) [32]. However, the reported ages of diagnosing mammary neoplasia in three studies from India, Bhutan and Malaysia were 7–9 years [33], 8.4 years [34] and 8.6 years [20] respectively. It is interesting to note that dogs from Asia have been reported to develop MGTs at an earlier age than dogs from North America, South America and Europe. Whether this reflects exposure to an external carcinogen, or a genetic predisposition in dogs in Asian counties is unknown. Other possible factors include different feeding practices, spaying practices, and immunization protocols followed in Asian countries compared to those of North American, South American and European countries [35]. Alternatively, it is possible that dogs in the Asian countries do not live long and the earlier onset of mammary neoplasia detected in these countries is simply due to fewer old dogs in these populations.

The proportion of dogs with multiple MGTs in this study (27.6%) was higher than the proportions of dogs reported in the majority of previous studies mainly of dogs from Western countries [33,36]. An exception was a Malaysian study in which 29.2% of dogs were reported to have multiple MGTs [20]. Multiple MGTs is well recognized in dogs [21], and is believed to be due to the concurrent exposure of all mammary glands in a single animal to circulating reproductive hormones. The resulting tumors are more likely to be at the same stage of development, and are more frequently benign than malignant [21]. However, benign to malignant transformation may occur in some tumors over time, resulting a combination of malignant and benign tumors in the same animal [21].

In the present study, mixed-breed dogs had the highest incidences of MGTs. Generally, mixed-breed dogs are considered to be comparatively less predisposed for MGTs compared to pure breeds [32]. In Sri Lanka, mixed-breed dogs are reportedly the most common pet dog breed [37]. Thus, the predominance of them in the present study is more likely to be a reflection of their commonality, rather than a true breed predisposition. Similarly, the over-representation of German shepherds in the present study might also be due to their high popularity in Sri Lanka, and may not necessarily indicate a breed predisposition. The minimal representation of Labradors in this study is noteworthy, considered the reportedly high popularity of this breed among Sri Lankan dog owners [35].

Most of the dogs in the present study had an ideal body condition score (BCS 3). Obesity at 1 year of age and in the year before the diagnosis of mammary neoplasia is significantly related with a higher prevalence of MGTs [38]. In contrast, we found only 5 obese dogs in our study. While this may suggest obesity does not predispose to MGTs, this cannot be determined without the knowledge of overall proportion of obese dogs in the population.

Previous studies have reported that dogs with MGTs are generally healthy at the initial presentation [35]. However, most of the dogs included in our study were systemically ill at the time of presentation, which might be due to tumor metastasis or other concurrent diseases. Systemic illness due to tumor metastasis was confirmed in few cases in the present study, due to the unavailability of necessary diagnostic testing. In systemically ill dogs, pre-surgical patient stabilization is important for successful surgical and post-surgical management [21]. Therefore, the results suggest that Sri Lankan veterinary surgeons should be more vigilant about the pre-surgical patient stabilization procedures to minimize the possible post-surgical complications.

## 5. Conclusions

A majority of mammary gland tumors in Sri Lankan dogs were histologically malignant, and a considerably high histological diversity was observed. Tumor size and location were identified to be predictive of malignancy, even though they are not confirmative of malignancy when considered alone. Relatively younger dogs were found to be affected; the mean age of MGT diagnosis was 8.0 ± 2.41 years. Nulliparous dogs predominated in the group. Overall, the present findings emphasize the necessity of improving awareness on MGTs among Sri Lankan clinicians, as well as dog owners.

## Figures and Tables

**Table 1:** Reproductive status of the dogs with mammary gland tumors.

Reproductive Status	n of Cases	%
Intact	46	62.2
OHE		
OHE: ≤3 years	8	10.8
OHE: 4–6 years	12	16.2
OHE: ≥7 years	6	8.1
Unknown	2	2.7

**Table 2:** Distribution of mammary gland tumors.

Location	Benign	Malignant	Total
Thoracic	5 (55.6%)	9 (13.8%) ^a^	14 (18.9%)
Abdominal	2 (22.2%)	12 (18.4%) ^a^	14 (18.9%)
Inguinal	2 (22.2%)	44 (67.7%) ^b^	46(62.2%)
Total	9 (100%)	65 (100%)	74 (100%)

For malignant neoplasms, uncommon superscripts (a, b) between different tumor locations indicate significant differences. Critical and absolute values: Thoracic—Abdominal (0.13, 0.04), Thoracic—Inguinal (0.17, 0.55), Abdominal—Inguinal (0.17, 0.50).

**Table 3:** Size of mammary gland tumors.

	Benign	Malignant	Total
T1	8 (88.8%)	10 (15.4%) ^a^	18 (24.3%)
T2	1 (11.1%)	30 (47.7%) ^b^	31 (41.9%)
T3	0 (0%)	25 (38.5%) ^b^	25 (33.8%)
Total	9 (100%)	65 (100%)	74 (100%)

For malignant neoplasms, uncommon superscripts (a, b) between different tumor size categories indicate significant differences. Critical and absolute values: T1–T2 (0.18, 0.32), T1–T3 (0.17, 0.22), T2–T3 (0.2, 0.1).

**Table 4:** Histological sub-types of mammary gland tumors.

Malignant Tumors	65	n of Cases	%
**(a) Carcinomas**	45		
Carcinoma-simple		13	17.6
Carcinoma: mixed type		10	13.5
Carcinoma-solid		6	8.1
Intra-ductal papillary carcinoma		5	6.7
Comedocarcinoma		5	6.7
Carcinoma: complex		3	4.1
Ductal carcinoma		1	1.3
Carcinoma-anaplastic		1	1.3
Carcinoma in situ		1	1.3
**(b) Carcinomas: special types**	16		
Adenosquamous carcinoma		8	10.8
Squamous cell carcinoma		3	4.1
Lipid-rich carcinoma		2	2.7
Carcinoma-spindle cell variant		1	1.3
Inflammatory carcinoma		1	1.3
Malignant myoepithelioma		1	1.3
**(c) Sarcomas**	**4**		
Hemangiosarcoma		2	2.7
Fibrosarcoma		1	1.3
Osteosarcoma		1	1.3
**Benign Tumors**	**9**	**n of Cases**	**%**
Simple adenoma		3	4.1
Intra-ductal papilloma		2	2.7
Complex Adenoma		2	2.7
Fibroadenoma		1	1.3
Mixed-benign tumor		1	1.3
